# Nucleos(t)ide analogs for hepatitis B virus infection differentially regulate the growth factor signaling in hepatocytes

**DOI:** 10.1097/HC9.0000000000000351

**Published:** 2024-01-05

**Authors:** Ryogo Shimizu, Kazuhisa Murai, Kensuke Tanaka, Yuga Sato, Naho Takeda, Saki Nakasyo, Takayoshi Shirasaki, Kazunori Kawaguchi, Tetsuro Shimakami, Kouki Nio, Yuki Nakaya, Harumi Kagiwada, Katsuhisa Horimoto, Masashi Mizokami, Shuichi Kaneko, Kazumoto Murata, Taro Yamashita, Masao Honda

**Affiliations:** 1 Department of Clinical Laboratory Medicine, Kanazawa University Graduate School of Medical Sciences, Kanazawa, Japan; 2 Department of Gastroenterology, Kanazawa University Graduate School of Medicine, Kanazawa, Japan; 3 Department of Infection and Immunity, Division of Virology, Jichi Medical University, Shimotsuke, Japan; 4 Biological Data Science Research Group, Cellular and Molecular Biotechnology Research Institute, National Institute of Advanced Industrial Science and Technology, Tokyo, Japan; 5 Artificial Intelligence Research Center, National Institute of Advanced Industrial Science and Technology, Tokyo, Japan; 6 Genome Medical Sciences Project, National Center for Global Health and Medicine, Ichikawa, Japan

## Abstract

**Background:**

Recent clinical studies have suggested that the risk of developing HCC might be lower in patients with chronic hepatitis B receiving tenofovir disoproxil fumarate than in patients receiving entecavir, although there is no difference in biochemical and virological remission between the 2 drugs.

**Methods:**

The effects of nucleoside analogs (NsAs; lamivudine and entecavir) or nucleotide analogs (NtAs; adefovir disoproxil, tenofovir disoproxil fumarate, and tenofovir alafenamide) on cell growth and the expression of growth signaling molecules in hepatoma cell lines and PXB cells were investigated *in vitro*. The tumor inhibitory effects of NsAs or NtAs were evaluated using a mouse xenograft model, and protein phosphorylation profiles were investigated. The binding of NsAs or NtAs to the insulin receptor (INSR) was investigated by thermal shift assays.

**Results:**

NtAs, but not NsAs, showed direct growth inhibitory effects on hepatoma cell lines *in vitro* and a mouse model *in vivo*. A phosphoprotein array revealed that INSR signaling was impaired and the levels of phosphorylated (p)-INSRβ and downstream molecules phosphorylated (p)-IRS1, p-AKT, p-Gab1, and p-SHP2 were substantially reduced by NtAs. In addition, p-epidermal growth factor receptor and p-AKT levels were substantially reduced by NtAs. Similar findings were also found in PXB cells and nontumor lesions of liver tissues from patients with chronic hepatitis B. Prodrug NtAs, but not their metabolites (adefovir, adefovir monophosphate, adefovir diphosphate, tenofovir, tenofovir monophosphate, and tenofovir diphosphate), had such effects. A thermal shift assay showed the binding of NtAs to INSRβ.

**Conclusions:**

NtAs (adefovir disoproxil, tenofovir disoproxil fumarate, and tenofovir alafenamide), which are adenine derivative acyclic nucleotide analogs, potentially bind to the ATP-binding site of growth factor receptors and inhibit their autophosphorylation, which might reduce the risk of HCC in patients with chronic hepatitis B.

## Graphical abstract

**Figure:**
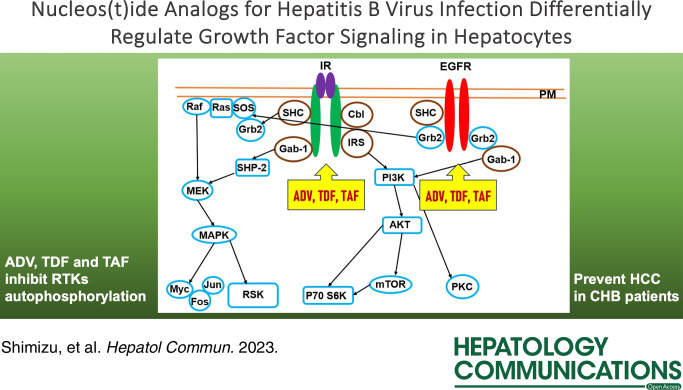

## INTRODUCTION

(HBV infection is the major cause of HCC, and 250 million people worldwide are chronic carriers of HBV.^1^ Nucleos(t)ide analogs (NAs) have been used widely to suppress HBV replication in patients with chronic hepatitis B (CHB) and prevent the onset of HCC.^2^ To date, entecavir (ETV) and tenofovir disoproxil fumarate (TDF), along with the recently introduced tenofovir alafenamide (TAF),^3^ have been commonly used as the first-line antiviral drugs in patients with CHB due to the high genetic barriers to drug resistance.

Nucleotide analogs [NtAs; adefovir disoproxil (ADV) and TDF], but not nucleoside analogs [NsAs; lamivudine (LMD) and ETV], possess an additional pharmacological effect by inducing the production of interferon (IFN)-λ3, which further induces the expression of IFN-stimulated genes and reduces HBV surface antigen production.^4,5^ It was reported that NtAs induce IFN-λ3 expression in colon cancer cells but not in hepatocyte cell lines (HepG2, Huh7, and PXB cells).^4^ Therefore, physiologically, IFN-λ3 could be induced in gut epithelial cells and then transported to the liver through the portal vein and have effects on hepatocytes, thereby reducing HBV replication in patients with CHB.^4^

In addition to these findings, recent clinical studies have reported that the risk of developing HCC is significantly lower in patients with CHB receiving TDF than in patients receiving ETV,^6,7^ although these findings are controversial.^8,9^ ETV-treated and TDF-treated patients have similar rates of on-therapy biochemical and virological remission, HBV surface antigen loss, liver transplantation, and/or death.^8^ Therefore, the precise mechanisms by which TDF reduces the risk of HCC in patients with CHB are unknown.

In this study, we found that NtAs and NsAs have differential effects on hepatocytes by affecting growth factor receptor signaling. These findings provide an alternative mechanism by which TDF, rather than ETV, can prevent HCC.

## METHODS

### Study approval and ethics statements

Animal experiments were approved by the Ethics Committee for the Care and Use of Laboratory Animals at the Takara-Machi Campus of Kanazawa University, Japan, and were carried out in compliance with the ARRIVE guidelines 2.0. All experiments were performed in accordance with relevant guidelines and regulations. For human participants, the research protocols were conducted in accordance with the Declarations of Helsinki and Istanbul, and approved by the Human Genome/Gene Analysis Research Ethics Committee of Kanazawa University and its related hospitals. Written informed consent was obtained from all patients.

### Cell culture and reagents

Cells were maintained in DMEM (Thermo Fisher Scientific, Waltham, MA) containing 10% fetal bovine serum, 100 U/mL penicillin, and 100 mg/mL streptomycin and maintained at 37°C with 5% CO_2_. PXB cells (female, 2 years) were purchased from PhoenixBio (Hiroshima, Japan). The following NAs were used: ETV (TOCRIS, Tokyo, Japan), ADV (Adooq Bioscience, Irvine, CA), adefovir (AFV; Selleckchem, Houston, TX), adefovir monophosphate (AFV-MP; Santiago, Redwood City, CA), adefovir diphosphate (AFV-DP; Moravek Inc., Brea, CA), TDF (Adooq Bioscience), TAF (Selleckchem), tenofovir (TFV; Selleckchem), tenofovir monophosphate (TFV-MP; Santiago), and tenofovir diphosphate (TFV-DP; Biomol, Hamburg, Germany).

The glucose concentrations of cell supernatants were measured using Glucose Assay Kit-WST (Dojindo, Kumamoto, Japan), and the glycogen concentrations in cells were measured using Glycogen Assay Kit (Abcam, Cambridge, UK).

### Xenograft model

NOD-SCID mice were purchased from the Jackson Laboratory. Six-week-old male mice were used for the study. DMSO, ETV, ADV, and TDF were administered intraperitoneally to the mice at a concentration of 90 mg/kg every 3 days. At 7 days after the first treatment with the NAs, the mice were injected subcutaneously with 5.0 × 10^6^ HepG2 cells in the right flank. At day 50, tumor volume was evaluated.

### Phosphorylation protein arrays

Phosphorylation activity was measured as described.^10,11^ Briefly, 1373 signal transduction proteins with a glutathione S-transferase-tag were synthesized and loaded onto a glutathione-coated glass slide. Every protein was spotted (~500 nL per spot) 6 times (n = 6) per slide, while ensuring they did not denature or dry. During spotting, the humidity was maintained at 40%–60%, and the wetness of each drop was confirmed by microscopic observation of the liquid droplets. Cell lysates of 100 μg total protein were applied to the array with additional ATP at 30°C for 3 hours. After termination of the kinase reaction, the array was washed with Tris-buffered saline containing 0.05% Tween 20 and stained with a 4G10 phosphorylated protein-specific antibody (Merck, #05-1050) and a secondary fluorescein-conjugated antibody (Thermo, #A21235) to detect the phosphorylated tyrosine residues. Signal data were filtered and normalized by BRB-ArrayTools (https://brb.nci.nih.gov/BRB-ArrayTools) as described.^12^ Differentially expressed phosphorylated proteins were obtained using a class comparison tool. Functional ontology enrichment analysis was conducted to compare the BioCarta Pathway process distribution of the differentially expressed genes. LS/KS permutation tests were performed for pathway comparison (*p* < 0.05) (BRB-ArrayTools; https://brb.nci.nih.gov/BRB-ArrayTools).

### qRT-PCR

Total RNA was isolated using a GenElute Mammalian Total RNA Miniprep Kit (Sigma-Aldrich, St. Louis, MO), and cDNA was synthesized using a High-Capacity cDNA Reverse Transcription Kit (Applied Biosystems, Carlsbad, CA). qRT-PCR was performed on the 7500 Real-Time PCR System (Applied Biosystems), using the primer pairs and probes for glucose-6-phosphatase catalytic subunit 1 (*G6PC1*), phosphoenolpyruvate carboxykinase 1 (*PCK1*), and *ACTB*, which were obtained from the TaqMan assay reagents library.

### Quantification of HBV-DNA

An HBV Quantitative Measurement Kit Ver. 2 (KUBIX) was used to extract HBV-DNA from the cell culture supernatant. HBV-DNA was quantified by qPCR using the following primer set: forward, 5′-ACTCACCAACCTCCTGTCCT-3′; reverse, 5′-GACAAACGGGCAACATACCT-3′; TaqMan probe, 5′-(FAM)-TATCGCTGGATGTGTCTGCGGCGT-(MGB-NFQ)-3′. A QuantStudio 3 Real-Time PCR System was used to detect HBV-DNA.

### Western blotting

The cells were lysed in a 1× RIPA Lysis Buffer (EMD Millipore, Burlington, MA) containing a complete Protease Inhibitor Cocktail and PhosSTOP (Roche Applied Science, Penzberg, Germany). Proteins were electrophoresed on 4%–20% Mini-PROTEAN TGX Precast Gels (Bio-Rad, Hercules, CA) and transferred to membranes using a Trans-Blot Turbo Transfer Pack PVDF (Mini) (Bio-Rad). The membranes were blocked with Can Get Signal/PVDF Blocking Reagent (Toyobo, Osaka, Japan) and incubated with primary antibodies overnight using Can Get Signal Solution 1 (Toyobo). After washing three times with Tris-buffered saline containing 0.05% Tween 20, the membranes were incubated with HRP-conjugated anti-rabbit or anti-mouse IgG (Cell Signaling Technology, Danvers, MA) for 1 hour. The membranes were imaged by ChemiDoc XRS Plus (Bio-Rad). The following primary antibodies were used: phosphorylated (p)-epidermal growth factor receptor (EGFR; Tyr1068) (D7A5), EGFR (D38B1), phosphorylated (p)-IGF-I receptor β (Tyr1135/1136)/insulin receptor β (INSRβ; Tyr1150/1151) (19H7), IGF-I receptor β, p-AKT (Ser473), AKT (pan) (11E7), p-SHP-2 (Tyr542), SHP-2 (D50F2), β-actin, and GAPDH (Cell Signaling Technology).

### Protein thermal shift assay

Protein Thermal Shift Software ver. 1.4 was purchased from Applied Biosystems and installed into QuantStudio 3 (Applied Biosystems). INSR active protein (SignalChem Biotech Inc., Richmond, BC) was incubated with or without 100 µM NA ligand (ETV, ADV, TAF, or TDF) in a buffer containing 20 mM Tris-HCl (pH 8.0), 50 mM NaCl, and 1 mM DTT. Proteins were stained with 60× SYPRO Orange (Sigma-Aldrich). The change of melting temperature (Tm) by NAs was calculated from the melt curve using Protein Thermal Shift Software.

### Statistical analysis

Statistical analysis was carried out by one-way ANOVA or a two-sided *t* test. Calculations were performed using Prism 8 software (GraphPad Software, La Jolla, CA).

## RESULTS

### NtAs and NsAs have differential effects on cell growth signaling in hepatoma cells

**FIGURE 1:**
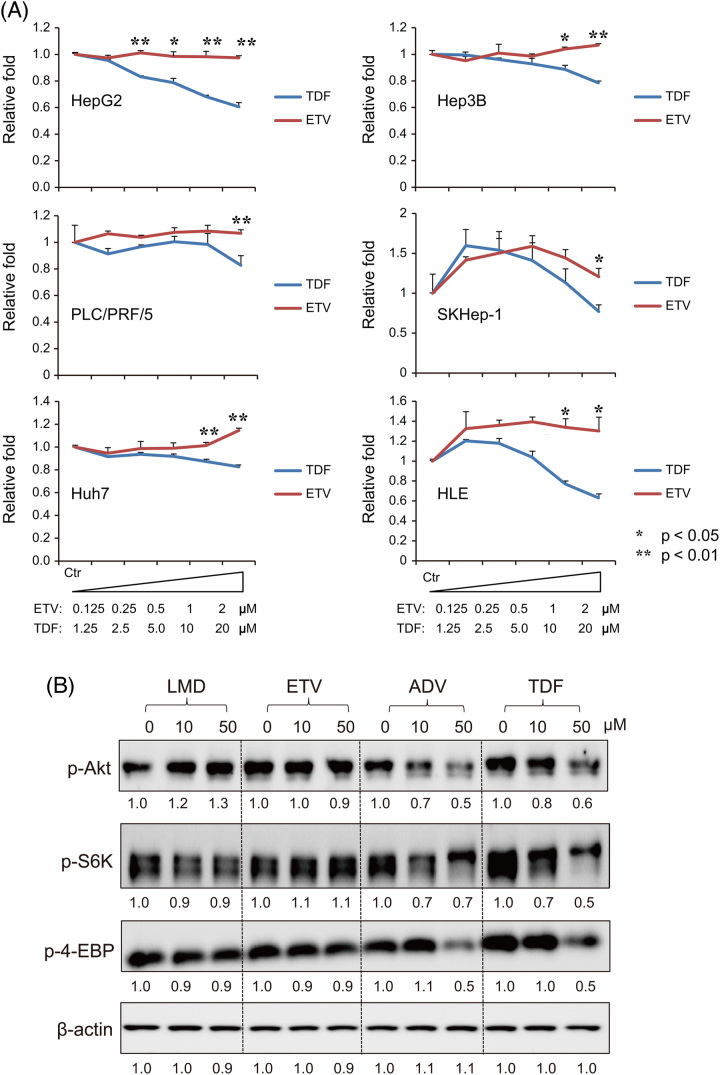
Nucleoside analogs (NsAs) and nucleoside analogs (NtAs) have differential effects on the growth of hepatocytes. (A) Six hepatoma cell lines (HepG2, Hep3B, PLC/PRF/5, SKHep-1, Huh7, and HLE cells) were treated with increasing concentrations of ETV (NsA; 0.125–2 μM) and TDF (NtA; 1.25–20 μM). Cell growth was measured using an ATP assay. (B) HepG2 cells were incubated with increasing concentrations (0, 10, 50 μM) of LMD, ETV, ADV, and TDF. p-AKT, p-S6K, and p-4EBP levels were evaluated after 24 hours. Abbreviations: ADV, adefovir disoproxil; ETV, entecavir; LMD, lamivudine; TDF, tenofovir disoproxil fumarate.

To examine the effects of NtAs and NsAs on hepatocytes, the impact of TDF (NtA) and ETV (NsA) on the cell growth of 6 hepatoma cell lines was investigated. At 1 day after the cells were passaged, TDF or ETV was added at different concentrations, and after 7 days, the surviving cells were measured by an ATP assay (Figure 1A). Interestingly, TDF-treated cells showed significantly lower survival than ETV-treated cells, especially at high concentrations of TDF.

We next examined the effects of NtAs and NsAs on the expression of growth signaling molecules in HepG2 cells (Figure 1B). Interestingly, NtAs (ADV and TDF) reduced the levels of p-ATK, p-S6K, and p-4EBP in a dose-dependent manner, whereas NsAs (LMD and ETV) had no effect on their levels (Figure 1B). These results suggest that NtAs might repress cell growth signaling, while NsAs may not.

### Suppression of tumor growth by NtAs in a xenograft tumor model

**FIGURE 2:**
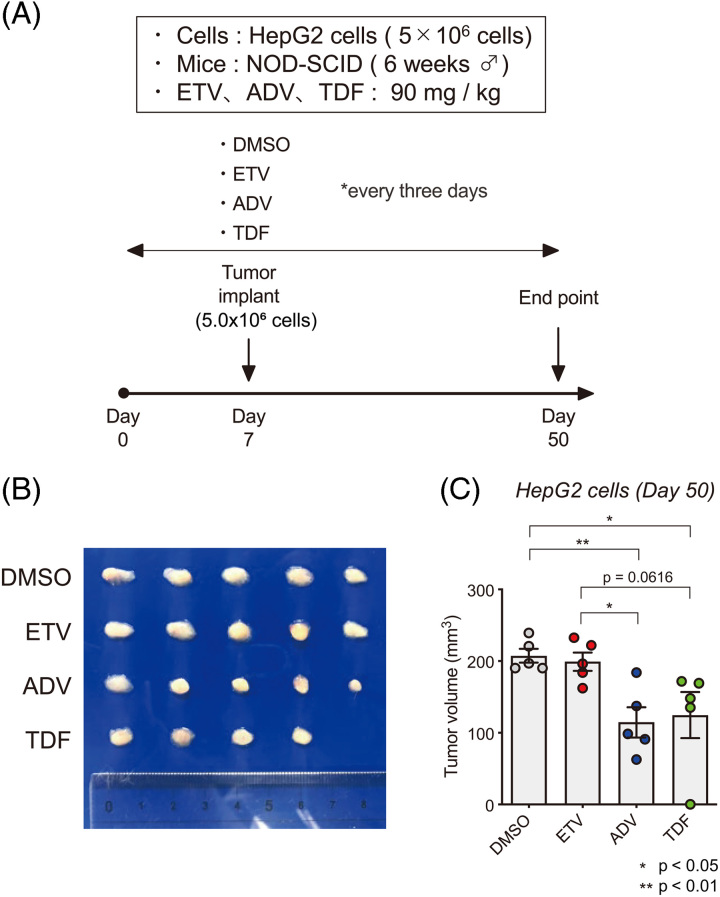
Antitumor effects of nucleos(t)ide analogs. (A) Experimental schedule of nucleos(t)ide analogs administration to tumor-bearing mice. DMSO, ETV, ADV, and TDF were administered intraperitoneally to NOD-SCID mice at a concentration of 90 mg/kg every 3 days. At 7 days after the initial treatment, HepG2 cells were implanted into the mice. On day 50, tumor volume was evaluated. (B) Photographs of the tumors excised on day 50. (C) Volume of the tumors excised on day 50. Abbreviations: ADV, adefovir disoproxil; ETV, entecavir; TDF, tenofovir disoproxil fumarate.

**FIGURE 3:**
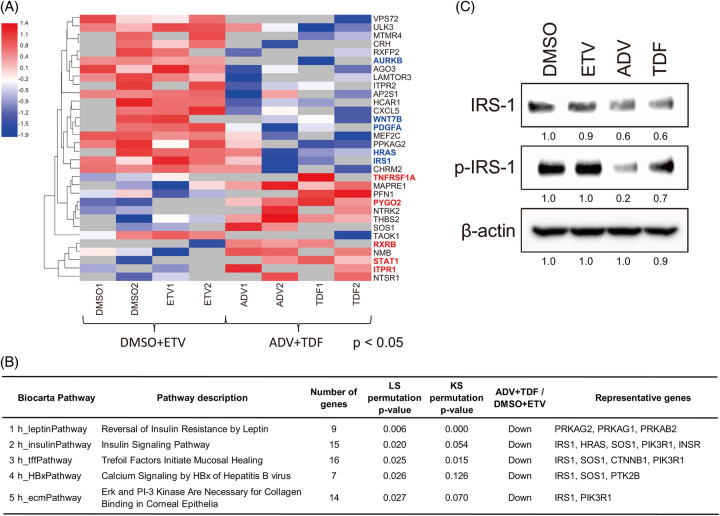
Phosphorylated protein profiles of xenografted tumors treated with nucleos(t)ide analogs. (A) Heat map of differentially expressed phosphorylated proteins (*p*<0.05) of xenografted tumors treated with DMSO+ETV or ADV+TDF. (B) Functional ontology enrichment analysis to compare the BioCarta Pathway process distribution of the differentially expressed genes. LS/KS permutation tests were performed for pathway comparison (*p*<0.05). (C) IRS-1 and p-IRS-1 levels of xenografted tumors treated with DMSO, ETV, ADV, and TDF. Abbreviations: ADV, adefovir disoproxil; ETV, entecavir; INSR, insulin receptor; IRS-1, insulin receptor substrate 1; TDF, tenofovir disoproxil fumarate.

To examine the inhibitory effects of NtAs on cell growth *in vivo*, a xenograft model of HepG2 cells in NOD-SCID mice was established (Figure 2). NtAs (ADV or TDF), NsA (ETV), or DMSO (solvent) were injected intraperitoneally every 3 days from day 0 to day 50. Tumor cells were implanted into the subcutaneous space on day 7, and the tumors were evaluated on day 50 (Figure 3A). Although the differences were not significant, tumor size was smaller in ADV-treated or TDF-treated mice than in ETV-treated or control DMSO-treated mice (Figure 2B). Tumor volume was significantly smaller in ADV-treated or TDF-treated mice than in DMSO-treated mice. There was a marginal difference in tumor volume between ETV-treated and TDF-treated mice (*p* = 0.0616). Thus, NtAs had a tumor-suppressive effect on hepatoma cells *in vitro* and *in vivo*.

### Examination of protein phosphorylation status of xenografted tumors by a novel protein array platform

We previously developed a platform for protein array phosphorylation measurement and pathway analysis.^10^ The protein array contained 1373 native proteins of 376 “pathway maps,” which are assigned as “signal transduction” pathways in the Reactome public database.^11^ We measured the phosphorylation activity of xenografted tumors using this native protein array and compared the phosphorylation status between the DMSO+ETV and ADV+TDF groups (Figure 3A). Thirty-two proteins were differentially phosphorylated (*p*<0.05) between both groups; the heat map of those proteins is shown in Figure 3A. Pathway comparison analysis showed that the insulin signaling pathway was the most differentially regulated between both groups (Figure 3B). Therefore, we focused on insulin signaling and validated the protein expression of insulin receptor substrate 1 (IRS-1), which is phosphorylated by INSR tyrosine kinase and plays an important role in insulin signaling. p-IRS-1 levels were reduced in the ADV+TDF group compared with the DMSO+ETV group (Figure 3C).

### Effects of NtAs on INSR signaling in HepG2 cells

**FIGURE 4:**
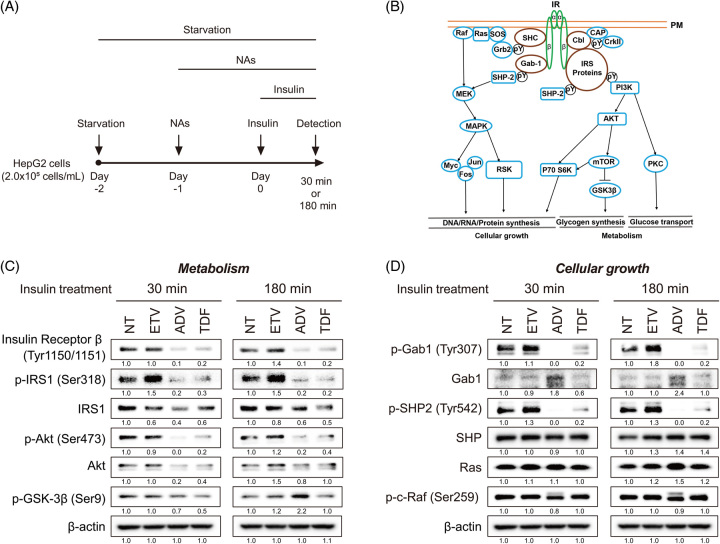
Effects of NAs on INSR signaling. (A) Experimental schedule of NA and insulin treatment. HepG2 cells were serum-starved for 24 hours and then pretreated with 50 μM of each NA. At 24 hours after NA treatment, the cells were treated with 100 μM insulin for 30 or 180 minutes. (B) Schematic diagram depicting the metabolism and cellular growth signaling pathways. (C, D) The effects of NAs on the insulin receptor–-mediated metabolism (C) and cellular growth (D) signaling pathways evaluated by western blotting. Abbreviations: IRS, insulin receptor substrate; NAs, nucleos(t)ide analogs; NT, Non nucleos(t)ide analogs treatment.

To determine the target molecules of NtAs (ADV and TDF) in insulin signaling, we evaluated the detailed effects of NtAs on INSR signaling in HepG2 cells (Figure 4A). ETV, ADV, or TDF was added to HepG2 cells at 1 day before insulin stimulation, and the cells were harvested at 30 or 180 minutes after insulin stimulation (Figure 4A). INSR is a disulfide-linked (αβ)2 homodimer that meditates metabolic (glycogen synthesis and glucose transport) and cell growth signaling (Figure 4B).^13^ Interestingly, INSRβ phosphorylation was substantially reduced in the ADV-treated and TDF-treated groups compared with the nontreatment and ETV-treated groups (Figure 4C). The levels of p-IRS1, a direct downstream molecule of p-INSRβ, were similarly reduced in the ADV-treated and TDF-treated groups (Figure 4C). In addition, PI3K-ATK signaling, located downstream of p-IRS1, was downregulated in the ADV-treated and TDF-treated groups (Figure 4B, C). Moreover, the levels of cell growth signaling molecules, p-Gab1, a direct downstream target of p-INSRβ, and p-SHP2, a downstream target of p-Gab1, were substantially reduced in the ADV-treated and TDF-treated groups (Figure 4D). These results indicate that NtAs inhibit INSR signaling pathways.

### TAF inhibits INSRβ and EGFR phosphorylation

**FIGURE 5:**
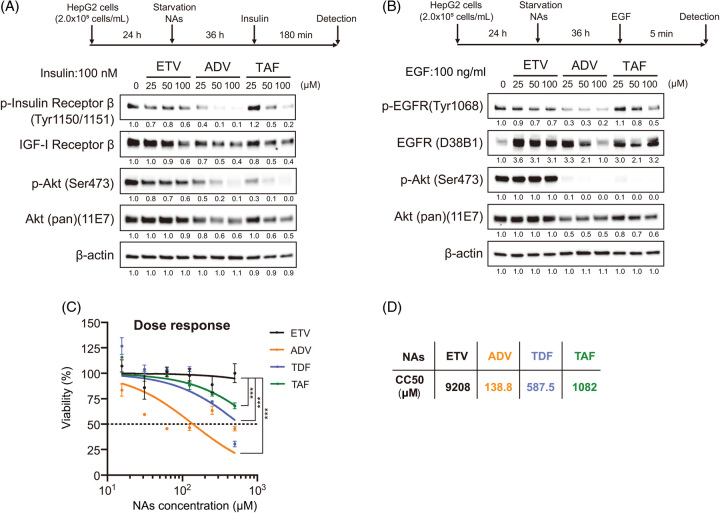
Dose-dependent effect of NAs on INSR and EGFR signaling. (A, B) HepG2 cells were serum-starved and pretreated with NAs at the indicated concentrations for 36 hours and then treated with 100 μM insulin for 180 minutes (A) or 100 ng/mL EGF for 5 minutes (B). Signaling pathways via insulin receptor and EGFR were evaluated by western blotting. (C) Cytotoxicity of NAs in HepG2 cells. HepG2 cells were treated with NAs at different concentrations (15.625, 31.25, 62.5, 125, 250, and 500 μM) for 72 hours. Cell viability was determined by cytotoxicity assays using a Cell Counting Kit-8. (D) CC50 was calculated by GraphPad Prism software. Abbreviations: ADV, adefovir disoproxil; EGFR, epidermal growth factor receptor; ETV, entecavir; NAs, nucleos(t)ide analogs; TAF, tenofovir alafenamide; TDF, tenofovir disoproxil fumarate.

TAF, a recently developed phosphonamide prodrug of TFV, is hydrolyzed to TFV in hepatocytes.^14,15^ TAF is formulated to deliver the active form found in hepatocytes more efficiently, with less than one-tenth the dose of TDF.^14,15^ Thus, lower serum concentrations provide similar efficacy without long-term kidney-related and bone-related side effects.^14,15^ We found that TAF inhibited INSR signaling in a dose-dependent manner, similar to ADV (Figure 5A). The INSR signaling pathway is essential for cell growth in hepatocytes; therefore, we reasoned it was important to evaluate other growth factor signaling pathways. EGF is a representative growth factor required for the regeneration of hepatocytes.^16^ Interestingly, ADV and TAF reduced the levels of p-EGFR and p-AKT (Figure 5B), while ETV had no effect on these molecules. Thus, NtAs might affect the growth factor signaling pathways other than INSR signaling. Among these NtAs, ADV was the most effective (CC50 = 138.8 μM) and efficiently suppressed INSR and EGFR signaling.

### Effects of NtAs on insulin-mediated glucose regulations in HepG2 cells

The liver contributes to the maintenance of blood sugar levels by producing glucose (gluconeogenesis) under fasting conditions. Insulin suppresses the expression of *G6PC1*, which induces the production of glucose by catalyzing the hydrolysis of D-glucose 6-phosphate to D-glucose. In addition, insulin suppresses the expression of *PCK1*, which induces glucose production in the TCA cycle by catalyzing the formation of phosphoenolpyruvate from oxaloacetate.

In this study, insulin treatment substantially repressed the expression of *G6PC1*. Meanwhile, cells treated with NtAs (ADV, TDF, and TAF) had a higher expression of *G6PC1* compared with non-NA-treated cells or ETV-treated cells (Supplemental Figure S1A, left, http://links.lww.com/HC9/A710). Moreover, insulin treatment repressed the expression of *PCK1* in non-NA-treated or ETV-treated cells. In contrast, the expression of *PCK1* was not suppressed or was even increased in the cells treated with NtAs (ADV, TDF, and TAF) (Supplemental Figure S1A, right, http://links.lww.com/HC9/A710).

Insulin treatment reduced the glucose concentrations in cell supernatants in non-NA-treated or ETV-treated cells. In contrast, glucose concentrations were increased in cells treated with NtAs (ADV, TDF, and TAF) (Supplemental Figure S1B, left, http://links.lww.com/HC9/A710). Correlated with these results, the glycogen contents in cells were increased by the insulin treatment in non-NA-treated or ETV-treated cells, whereas they were rather decreased in cells treated with NtAs (ADV, TDF, and TAF) (Supplemental Figure S1B, right, http://links.lww.com/HC9/A710). Thus, NtAs showed negative effects on insulin-mediated glucose regulation in HepG2 cells.

### Effects of NtAs on PXB cells and the liver of patients with CHB

**FIGURE 6:**
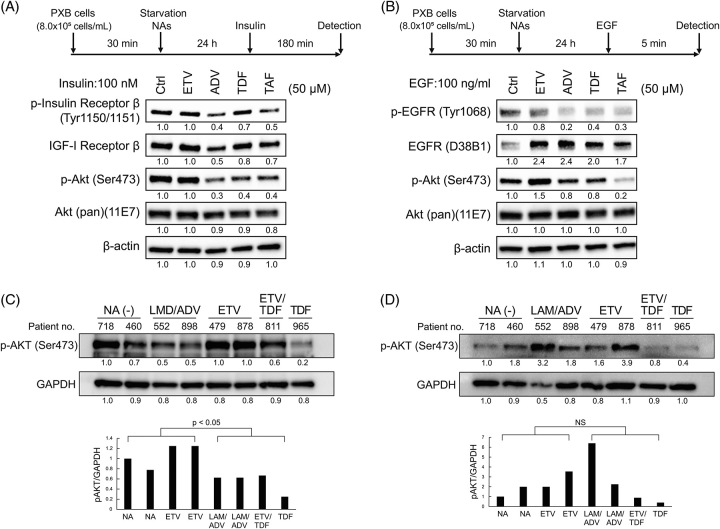
Effects of NAs on insulin receptor and EGFR signaling in PXB cells and p-AKT levels in liver tissues from patients with CHB. (A, B) PXB cells were serum-starved and pretreated with 50 μM NAs for 24 hours and then treated with 100 μM insulin for 180 minutes (A) or 100 ng/mL EGF for 5 minutes (B). Signaling pathways via INSR and EGFR were evaluated by western blotting. (C, D) p-AKT levels in nontumor (C) and tumor lesions (D) in the liver of patients with CHB complicated with HCC. Densitometric analysis of p-ATK levels in nontumor and tumor lesions is summarized in the lower panels. Abbreviations: ADV, adefovir disoproxil; EGFR, epidermal growth factor receptor ETV, entecavir; LMD, lamivudine; NAs, nucleos(t)ide analogs; NS, not significant; TAF, tenofovir alafenamide; TDF, tenofovir disoproxil fumarate.

We examined the effects of NtAs (ADV, TDF, and TAF) on PXB cells. PXB cells were incubated with ETV, ADV, TDF, or TAF for 24 hours and then stimulated with insulin or EGF (Figure 6). The levels of p-INSRβ and p-AKT in insulin signaling were reduced in NtA-treated PXB cells compared with those treated with ETV or DMSO, although TDF was less effective at reducing p-INSRβ levels (Figure 6A). In addition, the levels of p-EGFR and p-AKT in EGF signaling were reduced in NtA-treated PXB cells compared with those treated with ETV or DMSO, although the reduction of p-AKT by ADF or TDF was minimal (Figure 6B). These results indicate that NtAs repress the cell growth signaling pathways in PXB cells as well as in hepatoma cells, although the degree of repression by each molecule is slightly less in PXB cells than in hepatoma cells.

Furthermore, we evaluated the effects of NtAs in the liver tissues of patients with CHB (Supplemental Table S1, http://links.lww.com/HC9/A710). Tumor and nontumor lesions were obtained from patients with CHB complicated with HCC, and p-ATK levels were evaluated (Figure 6C, D). p-ATK levels were significantly lower in nontumor lesions in patients treated with ADV or TDF than in patients treated with ETV or without NAs (Figure 6C lower). However, the p-ATK levels of tumor lesions varied among individual patients and there was no trend of lower p-AKT levels induced by ADV or TDF (Figure 6D lower).

### IFN-λ3 has no effect on INSR and EGFR signaling in hepatocytes

It was reported that NtAs induce IFN-λ3 expression in colon cell lines (WiDr and HT-29 cells), but not in hepatocyte cell lines (HepG2, Huh7, and PXB cells).^4^ Therefore, it is hypothesized that colon epithelial cell-derived IFN-λ3 has an effect on the liver through portal vein flow.^4^ Accordingly, we examined the effects of IFN-λ3 on INSR and EGFR signaling in hepatocytes. We found that different concentrations of IFN-λ3 (1–100 ng/mL) and different incubation times with IFN-λ3 (12 and 24 h) had no effect on INSR and EGFR signaling in HepG2 cells (Supplemental Figure S2, http://links.lww.com/HC9/A710). Thus, the effect of NtAs on INSR and EGFR signaling in hepatocytes is independent of IFN-λ3.

### Prodrug NtAs, but not their diphosphate or triphosphate form, inhibit insulin and EGFR signaling

Prodrug ADV, TDF, or TAF is metabolized to AFV or TFV in cells.^17,18^ AFV and TFV are in the monophosphate form and are subsequently phosphorylated to the diphosphate form (AFV-MP or TFV-MP) and triphosphate form (AFV-DP or TFV-DP) by cellular kinases.^18^ Then, the triphosphate form (AFV-DP or TFV-DP) is incorporated into reverse transcription during HBV replication and inhibits HBV replication.

**FIGURE 7:**
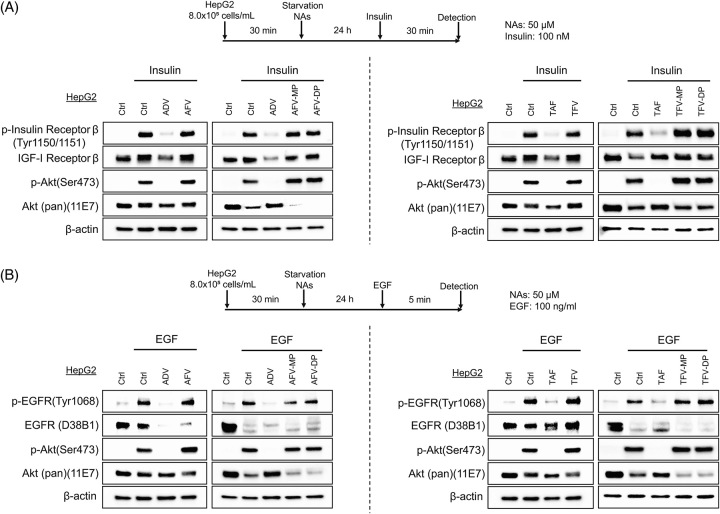
Effects of prodrug NAs and their metabolites (AFV, TFV, AFV-MP, AFV-DP, TFV-MP, and TFV-DP) on insulin receptor and EGFR signaling. (A) Experimental schedule of prodrug, metabolite, and insulin treatment. HepG2 cells were serum-starved and pretreated with 50 μM prodrug (ADV and TAF) or metabolite (AFV, TFV, AFV-MP, AFV-DP, TFV-MP, and TFV-DP) for 24 hours and then treated with 100 μM insulin for 30 minutes. Signaling pathways were evaluated by western blotting. (B) Experimental schedule of prodrug, metabolite, and EGF treatment. HepG2 cells were serum-starved and pretreated with 50 μM prodrug (ADV and TAF) or metabolites (AFV, TFV, AFV-MP, AFV-DP, TFV-MP, and TFV-DP) for 24 hours and then treated with 100 ng/mL EGF for 5 minutes. Signaling pathways were evaluated by western blotting. Abbreviations: AFV, adefovir; ADV, adefovir disoproxil; DP, diphosphate; EGFR, epidermal growth factor receptor; ETV, entecavir; MP, monophosphate; NAs, nucleos(t)ide analogs; TAF, tenofovir alafenamide; TDF, tenofovir disoproxil fumarate.

We compared the effects of prodrug (ADV or TDF; monophosphate), monophosphate form (AFV or TFV), diphosphate form (AFV-MP or TFV-MP), and triphosphate form (AFV-DP or TFV-DP) on the INSR or EGFR signaling pathways (Figure 7). We found that the monophosphate form (AFV or TFV), diphosphate form (AFV-MP or TFV-MP), and triphosphate form (AFV-DP or TFV-DP) did not inhibit INSR and EGFR signaling (Figure 7A and B).

To confirm that the prodrug metabolites could be incorporated into cells, we incubated HepG2.2.15 cells with AFV-MP, TFV-MP, or TFV-DP and examined their effects on HBV replication. AFV-MP, TFV-MP, and TFV-DP efficiently inhibited HBV replication as prodrugs (ADV and TDF; Supplemental Figure S3, http://links.lww.com/HC9/A710), implying that prodrug metabolites could be incorporated into cells. These results showed that prodrug NtAs, but not their metabolites, inhibit INSR and EGFR signaling.

### Direct interaction of NtAs with the INSRβ subunit

We further examined how NtAs inhibited INSR signaling. We first investigated the possibility that NtAs interfere with the binding of insulin to INSR. INSR consists of a disulfide-linked (αβ)2 homodimer, and insulin interacts mainly with the α-subunit, which is the extracellular domain. We performed a Biacore T200 assay to examine the direct interaction of NtAs (ADV, TDF, or TAF) with the α-subunit of INSR. We did not obtain any results showing the direct interaction of NtAs with the α-subunit of INSR, although insulin expectedly bound to this subunit (data not shown).

**FIGURE 8:**
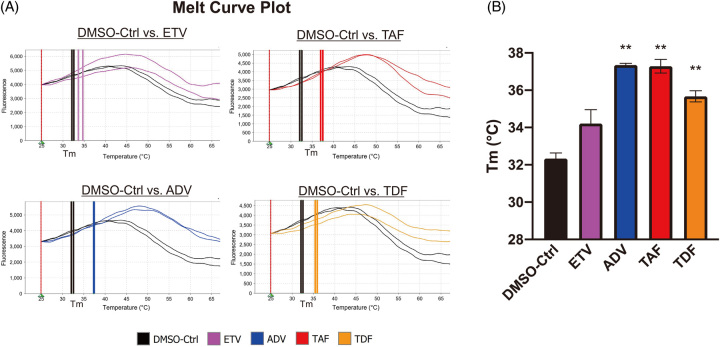
Direct effects of nucleos(t)ide analogs on insulin receptorβ thermal stability. (A) The thermal stability of INSR was determined by a protein thermal shift assay in the presence or absence of nucleos(t)ide analogs. Melt curve plots (Boltzmann method) were drawn by Protein Thermal Shift Software. (B) The change of Tm by nucleos(t)ide analogs was calculated from the melt curves using Protein Thermal Shift Software. Abbreviations: ADV, adefovir disoproxil; ETV, entecavir; TAF, tenofovir alafenamide; TDF, tenofovir disoproxil fumarate; Tm, melting temperature.

Then, we examined the complex formation of NtAs with the cytoplasmic β-subunit of INSR. We hypothesized that NtAs formed a complex with the β-subunit, thereby inhibiting its autophosphorylation. We utilized a thermal shift assay based on the finding that the binding of low-molecular-weight ligands can increase the thermal stability of a protein and Tm is shifted compared with no ligand-binding protein.^19^ Melt curve plots of the β-subunit of INSR combined with NtAs (ADV, TDF, or TAF) were obviously different from those with DMSO (Figure 8A). Tm (°C) was significantly higher in the presence of NtAs (ADV, TDF, and TAF) than with DMSO (Figure 8B). In contrast, there was no change in the melting curve for the β-subunit of INSR combined with ETV, and Tm (°C) was not significantly different from the DMSO control (Figure 8B). These results suggest that NtAs (ADV, TDF, and TAF) can bind to the β-subunit of INSR and interfere with its activation.

## DISCUSSION

NsAs (LMD and ETV) and NtAs (ADV, TDF, and TAF) have been utilized effectively to suppress HBV replication in patients with CHB and prevent the onset of HCC.^20^ From the point of view of clinical antiviral efficacy and safety and resistance to breakthrough mutations, the choice of NsAs and NtAs has been restricted to ETV and TDF or the new TFV derivative TAF. These drugs are structurally different and were developed independently.^21,22^ LMD and ETV are guanosine NsAs that contain guanine and sugar, while ADV, TDF, and TAF are AMP NtAs that contain adenine, an open ribose structure (acyclic), and a phosphate. It is a unique feature of ADV, TDF, and TAF that an open ribose acyclic structure is used instead of sugar.^22^

Clinical studies comparing the risk of HCC in patients with CHB who received TDF or ETV have been accumulating and a recent meta-analysis showed that TDF significantly lowers the risk of HCC compared with ETV.^7^ Although further clinical evaluations are needed to draw a definitive conclusion, so far, there has been no report showing that ETV is superior to TDF.^6–9^

In the present study, we demonstrated the direct growth inhibitory effects of NtAs on hepatoma cell lines *in vitro* (ADV, TDF, and TAF; Figure 1) and in a mouse xenograft model *in vivo* (ADV and TDF; Figure 2). By using a phosphoprotein array, we found that INSR signaling was impaired and the levels of p-INSRβ and its downstream molecules p-IRS1 p-AKT, p-Gab1, and p-SHP2 were substantially reduced by NtAs in HepG2 cells (Figures 3 and 4).

Interestingly, the suppression of the gluconeogenesis-related genes *G6PC1* and *PCK1* by the insulin treatment was significantly impaired by NtAs in HepG2 cells. Correlated with these results, insulin’s decreasing effects on glucose concentrations in cell supernatants and its increasing effects on glycogen contents in cells were significantly impaired by NtAs in HepG2 cells (Supplemental Figure S1, http://links.lww.com/HC9/A710).

In addition, we found that EGFR signaling was similarly impaired and the levels of p-EGFR and its downstream molecule p-AKT were substantially reduced by NtAs in HepG2 cells (Figure 5). Similar findings were also found in PXB cells (Figure 6A, B) and in the nontumor lesions of liver tissues from patients with CHB complicated with HCC (Figure 6C), although the reduction was lower in PXB cells than in HepG2 hepatoma cells.

Murata et al^4,5^ reported that NtAs (ADV and TDF), but not NsAs (LMD and ETV), induce IFN-λ3 expression in colon cancer cells and peripheral blood mononuclear cells. However, NtAs (ADV and TDF) do not induce IFN-λ3 expression in hepatoma cell lines and PXB cells.^4^ Therefore, it could be speculated that gut-produced IFN-λ3 might be carried through the portal vein and have effects on the liver. However, in our hands, IFN-λ3 added ectopically to the culture medium did not suppress INSR and EGFR signaling (Supplemental Figure S2, http://links.lww.com/HC9/A710). Thus, the effects induced by NtAs (ADV, TDF, and TAF) observed in the present study might be independent of the induction of IFN-λ3.

Prodrug ADV, TDF, or TAF is metabolized to AFV or TFV in cells and subsequently phosphorylated to the diphosphate form (AFV-MP or TFV-MP) and triphosphate form (AFV-DP or TFV-DP) by cellular kinases.^17,18^ The triphosphate form (AFV-DP or TFV-DP) is utilized to inhibit the reverse transcription of pregenomic HBV-RNA. Surprisingly, our results showed that prodrug NtAs (ADV, TDF, and TAF), but not their metabolites (AFV, TFV, AFV-MP, TFV-MP, AFV-DP, and TFV-DP), inhibited INSR and EGFR signaling.

How do prodrug NtAs (ADV, TDF, and TAF) affect the phosphorylation of the β-subunit of INSR and EGFR? We showed that NtAs (ADV, TDF, and TAF) form a complex with the β-subunit of INSR by using a thermal shift assay (Figure 8). ADV, TDF, and TAF contain adenine, an open ribose structure (acyclic), and a phosphate that shares some similarities with the structure of ATP (adenine, ribose, and three phosphates), while ETV contains guanine and ribose.^21,22^ It could be speculated that ADV, TDF, and TAF could bind to the ATP-binding site of the β-subunit of INSR and EGFR and inhibit its autophosphorylation. Further analysis should be performed to examine this hypothesis. In addition, other autophosphorylation sites such as AKT or SHP2 are potentially regulated by prodrug NtAs (ADV, TDF, and TAF). Additional studies are needed to determine which other signaling molecules are affected by prodrug NtAs.

There are several limitations in this study. First, the concentrations of NAs used for the cellular experiments were relatively high (up to 100 μM). Although it is difficult to estimate the appropriate concentrations of NAs for cellular experiments, according to the clinical dosage of ADV (10 mg), ETV (0.5 mg), TDF (300 mg), and TAF (25 mg), the physiological concentrations would be 4.3 μM for ADV, 0.37 μM for ETV, 103 μM for TDF, and 5.1 μM for TAF (for a patient weighing 60 kg with a blood flow of 4.6 L/min). Therefore, our experiments were performed using high concentrations of NAs, except TDF. Nevertheless, our study is useful when comparing patients with CHB who received ETV or TDF. Second, the effects of prodrug NtAs (ADV, TDF, and TAF) might be transient as they are metabolized to the final triphosphate form (AFV-DP or TFV-DP). We suppose the long duration of such mild effects might contribute to the reduction of the incidence of HCC without inducing severe side effects. However, recent studies showing that TDF-containing antiretroviral therapy in HIV-infected patients increases the risk of diabetes mellitus should be considered.^23,24^ It should be also noted that recent clinical studies have consistently reported that virological relapse after discontinuation of TDF occurs earlier than that after discontinuation of ETV.^25,26^ It has also been reported that the PI3K-AKT pathway plays important roles in suppressing HBV transcription;^27,28^ therefore, impairment of this signaling pathway by NtAs (ADV, TDF, and TAF) would potentiate the induction of HBV replication. Evaluation of insulin resistance in patients with CHB might be a good predictor for early relapse of HBV viremia after discontinuation of TDF.

In summary, we found that prodrug NtAs (ADV, TDF, and TAF) produce an additional pharmacological effect by inhibiting cell growth signaling pathways. This effect might be independent of the induction of IFN-λ3 expression reported previously.^4,5^ Our findings may partly reveal the unknown mechanism by which patients with CHB who receive TDF have a better prognosis and lower risk of developing HCC than the patients with CHB who receive ETV. Our data might contribute to the development of new chemopreventive drugs for HCC based on the detailed analysis of the structure of prodrug NtAs.

## Supplementary Material

SUPPLEMENTARY MATERIAL
