# Supplementary material for: Nucleos(t)ide analogs for hepatitis B virus infection differentially regulate the growth factor signaling in hepatocytes
Source: Hepatol Commun. 2024 Jan 5;8(1):e0351. doi: 10.1097/HC9.0000000000000351 (PMC10781114; doi:10.1097/HC9.0000000000000351)
Supplement: SUPPLEMENTARY MATERIAL [file hc9-8-e0351-s001.docx]

**Supporting Information for**

**Nucleoside and nucleotide analogs for hepatitis B virus infection differentially regulate growth factor receptor signaling in hepatocytes**

Ryogo Shimizu^1^, Kazuhisa Murai^1^, Kensuke Tanaka^1^, Yuga Sato^1^, Naho Takeda^1^, Saki Nakasyo^1^, Takayoshi Shirasaki^1^, Kazunori Kawaguchi^2^, Tetsuro Shimakami^2^, Kouki Nio^2^, Yuki Nakaya^3^, Harumi Kagiwada^4^, Katsuhisa Horimoto^5^, Masashi Mizokami^6^, Kazumoto Murata^3,6^, Taro Yamashita^2^, and Masao Honda^1,2^

Ryogo Shimizu and Kazuhisa Murai contributed equally to this work.

**Affiliation**

^1^ Department of Clinical Laboratory Medicine, Kanazawa University Graduate School of Medical Sciences, Kanazawa, Japan

^2^ Department of Gastroenterology, Kanazawa University Graduate School of Medicine, Kanazawa, Japan

^3^ Division of Virology, Department of Infection and Immunity, Jichi Medical University, Shimotsuke, Japan

^4^ Cellular and Molecular Biotechnology Research Institute, National Institute of Advanced Industrial Science and Technology, Tokyo, Japan

^5^ Artificial Intelligence Research Center, National Institute of Advanced Industrial Science and Technology, Tokyo, Japan

^6^ Genome Medical Sciences Project, National Center for Global Health and Medicine, Ichikawa, Japan

**Supplemental Table 1**

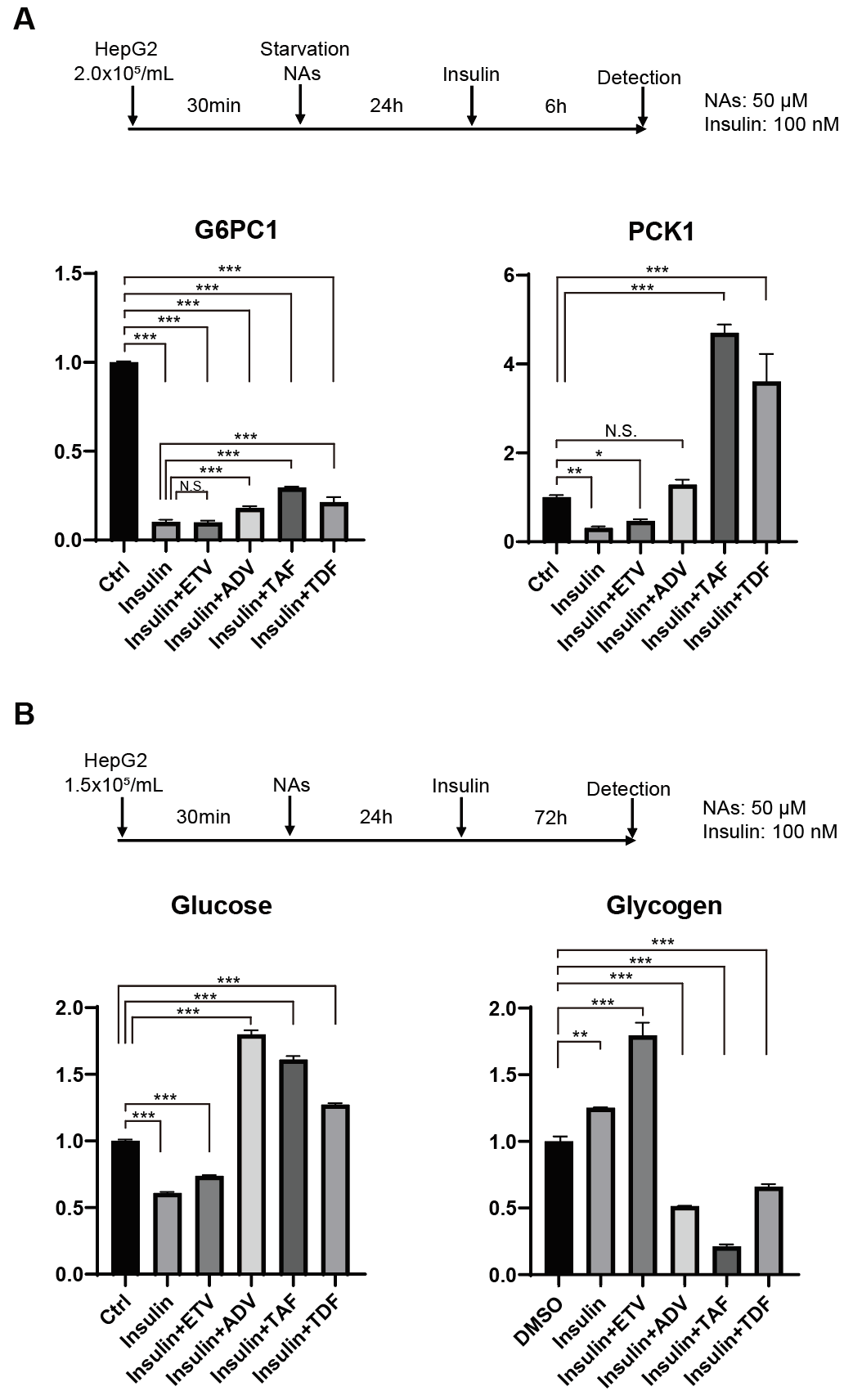
­

**Supplemental Figure 1.** Effects of NtAs on insulin mediated glucose regulations in HepG2 cells.

HepG2 cells were serum-starved and pre-treated with NAs at 50 μM for 24 h and then treated with 100 μM insulin. After 6 h, the expression of *G6PC1* and *PCK1* was detected by qRT-PCR (A). HepG2 cells were pre-treated with NAs at 50 μM for 24 h and then treated with 100 μM insulin. After 72 h, the glucose concentrations in cell supernatants and the glycogen concentrations in cells were measured (B).


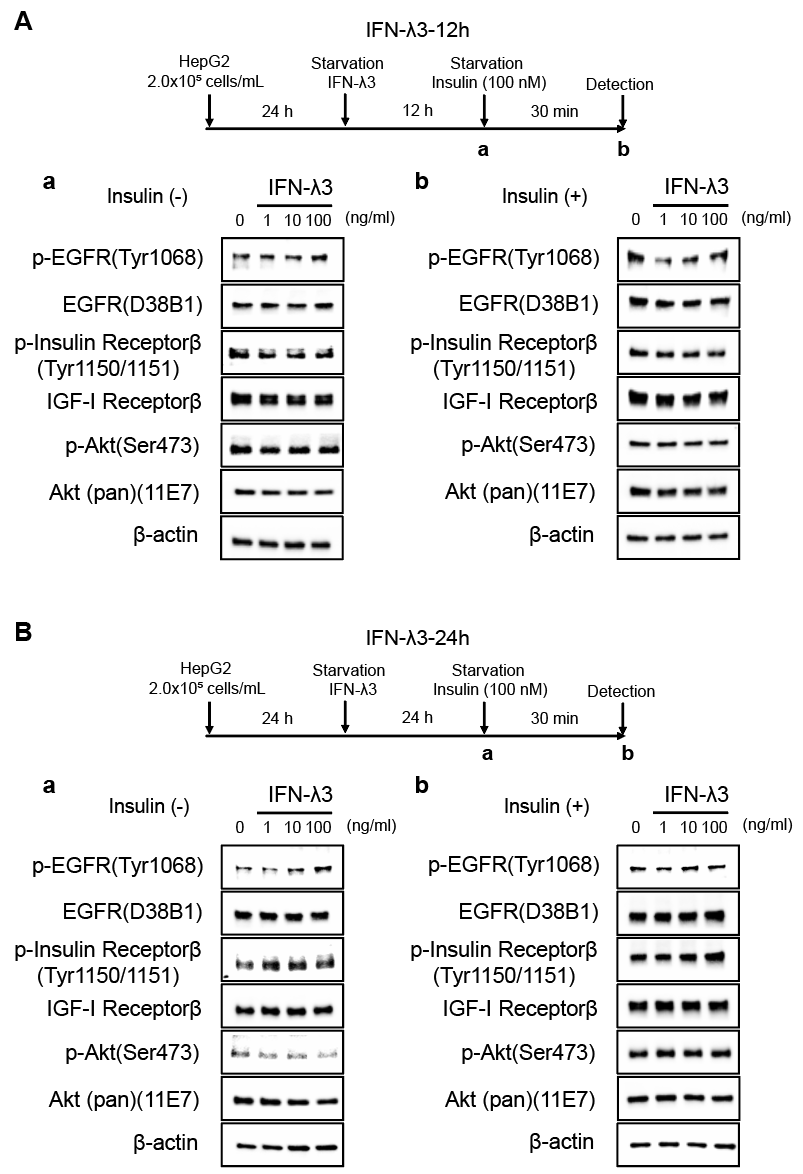


**Supplemental Figure 2.** Effects of IFN-λ3 on INSR and EGFR signaling.

HepG2 cells were serum-starved and treated with IFN-λ3 at the indicated concentration. At 12 (A) or 24 (B) h after treatment, the cells were incubated with or without 100 μM insulin for 30 min. The cells were analyzed by western blotting.


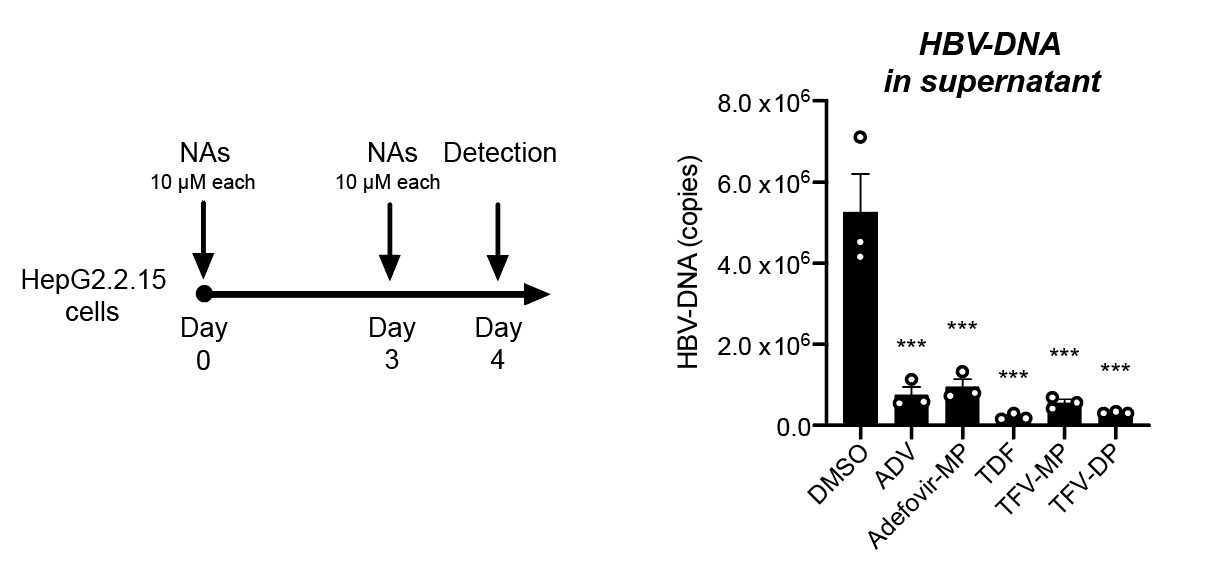


**Supplemental Figure 3.** Anti-HBV effects of NtAs and their metabolites.

HepG2.2.15 cells were treated with 10 μM NtAs and their metabolites at Day 0 and 3. At Day 4, the cell culture supernatant was collected, and HBV-DNA levels were measured by qPCR.
